# Comparison of Diagnostic Test Accuracy of Cone-Beam Breast Computed Tomography and Digital Breast Tomosynthesis for Breast Cancer: A Systematic Review and Meta-Analysis Approach

**DOI:** 10.3390/s22093594

**Published:** 2022-05-09

**Authors:** Temitope Emmanuel Komolafe, Cheng Zhang, Oluwatosin Atinuke Olagbaju, Gang Yuan, Qiang Du, Ming Li, Jian Zheng, Xiaodong Yang

**Affiliations:** 1 Department of Medical Imaging, Suzhou Institute of Biomedical Engineering and Technology, Chinese Academy of Sciences, Suzhou 215163, China or tekomolafe@shanghaitech.edu.cn (T.E.K.); zhangc@sibet.ac.cn (C.Z.); yuangang@sibet.ac.cn (G.Y.); dut@sibet.ac.cn (Q.D.); lim@sibet.ac.cn (M.L.); 2 School of Biomedical Engineering, ShanghaiTech University, Shanghai 201210, China; 3 Division of Life Sciences and Medicine, School of Biomedical Engineering (Suzhou), University of Science and Technology of China, Hefei 230026, China; 4 Molecular Imaging Research Center, Harbin Medical University, Harbin 150028, China; triplet852002@gmail.com; 5 TOF-PET/CT/MR Center, The Fourth Hospital of Harbin Medical University, Harbin 150028, China

**Keywords:** breast cancer, cone-beam computed tomography, digital breast tomosynthesis, meta-analysis, sensitivity, specificity

## Abstract

Background: Cone-beam breast computed tomography (CBBCT) and digital breast tomosynthesis (DBT) remain the main 3D modalities for X-ray breast imaging. This study aimed to systematically evaluate and meta-analyze the comparison of diagnostic accuracy of CBBCT and DBT to characterize breast cancers. Methods: Two independent reviewers identified screening on diagnostic studies from 1 January 2015 to 30 December 2021, with at least reported sensitivity and specificity for both CBBCT and DBT. A univariate pooled meta-analysis was performed using the random-effects model to estimate the sensitivity and specificity while other diagnostic parameters like the area under the ROC curve (AUC), positive likelihood ratio ($LR^{+}$), and negative likelihood ratio ($LR^{-}$) were estimated using the bivariate model. Results: The pooled sensitivity specificity, $LR^{+}$ and $LR^{-}$ and AUC at 95% confidence interval are 86.7% (80.3–91.2), 87.0% (79.9–91.8), 6.28 (4.40–8.96), 0.17 (0.12–0.25) and 0.925 for the 17 included studies in DBT arm, respectively, while, 83.7% (54.6–95.7), 71.3% (47.5–87.2), 2.71 (1.39–5.29), 0.20 (0.04–1.05), and 0.831 are the pooled sensitivity specificity, $LR^{+}$ and $LR^{-}$ and AUC for the five studies in the CBBCT arm, respectively. Conclusions: Our study demonstrates that DBT shows improved diagnostic performance over CBBCT regarding all estimated diagnostic parameters; with the statistical improvement in the AUC of DBT over CBBCT. The CBBCT might be a useful modality for breast cancer detection, thus we recommend more prospective studies on CBBCT application.

## 1. Introduction

Breast cancer is the most commonly diagnosed type of cancer among women that has led to the cause of cancer death in women of all ages [1,2]. This mortality rate can be reduced drastically if those cancers are detected early [1]. Digital mammography (DM) has been a conventional tool for early breast cancer diagnosis [3,4]. Recent research on both randomized controlled trials and observational studies has indicated that regular screening DM can reduce breast cancer drastically, which has a limitation of inability to image overlap dense breast tissue [5]. Digital breast tomosynthesis (DBT) has been developed to solve the tissue overlap of DM, and DBT acquisition involves an X-ray tube moving in an arc over the compressed breast taking multiple images from different angles. These images are reconstructed or synthesized into three-dimensional (3D) images via a reconstruction algorithm [6]. Several studies have recorded the improved diagnostic accuracy parameter such as sensitivity and specificity of 3D DBT alone or a combination with the DM [7,8,9,10]. A promising new technique is the dedicated cone-beam computed tomography (CBBCT) which provides real isotropic spatial resolution 3D images [6]. This modality also provides maximum breast comfortability to patients due to its reduced breast compression, unlike conventional DM and its DBT counterpart. Of particular importance is the CBBCT, which provides high-quality images and real-time 3D visualization of breast imaging and has proven to better visualize overlapping breast tissues than other imaging modalities like DM and ultrasound (US) [11,12,13]. Few studies have been documented on the review of diagnostic accuracy of DBT [14,15,16,17], while few pieces of literature have been recorded on the screening using CBBCT [18]. Contrast-enhanced cone-beam breast CT (CE-CBBCT) may improve the detection of breast cancer with possibly high specificity compared to that of DM, but with the cost of the high radiation exposure due to double scan. Uhlig et al. [19] carried out a meta-analysis study to compare the diagnostic performance of CE-CBBCT and that of non-contrast CBBCT (NC-CBBCT). They found a non-significant difference in sensitivity and specificity of CE-CBBCT, but considerable significance between-study heterogeneity in the NC-CBBCT.

Studies carried out about 10 years ago by Belair et al. [20] and Zuley et al. [21] compared the diagnostic accuracy of CBBCT and DBT, and their results showed that overall confidence in diagnosis was higher for both benign and malignant breast lesions using DBT. The authors suggested that future advances in technology and improvement in the readers’ performance might lead to better performance of CBBCT in the future. In the last 7 years, few studies have reported on the diagnostic accuracy of CBBCT, none of these studies has directly compared CBBCT with DBT or used a meta-analysis approach to address this issue by comparing the potential diagnostic ability of these two 3D breast imaging modalities is still a hanging fruit yet to plug. Therefore, this study aims to systematically review and analyze the diagnostic accuracy of existing studies on CBBCT and DBT for breast cancer detection, thereby increasing the statistical power and thus eliminating any disagreement between individual studies.

## 2. Materials and Methods

This systematic review and meta-analysis was prospectively registered at PROSPERO with the registration number of CRD: 42020180192 [22]. The systematic review was performed by two independent reviewers (TEK and OAO or CZ and GY) using a well-established review protocol adapted from the Cochrane collaborative approach for evaluating diagnostic test accuracy [23] with Preferred Reporting Items for Systematic Reviews and Meta-analyses (PRISMA) guidelines [24], see Supplementary File S1. The two reviewers discussed the discrepancies between the two results, and then a more experienced third reviewer (XY or JZ or ML) was consulted if the interrater consensus was not reached. We searched for women who underwent breast imaging screening using either CBBCT or DBT, which reported the characterization of malignant and benign lesions with well-documented diagnostic accuracy. We searched separately because no available literature reported comparison studies on CBBCT and DBT for diagnostic or screening purposes. This search includes comparative, prospective and retrospective studies, and interrater consensus.

### 2.1. Data Sources and Search Strategy

**Figure 1:**
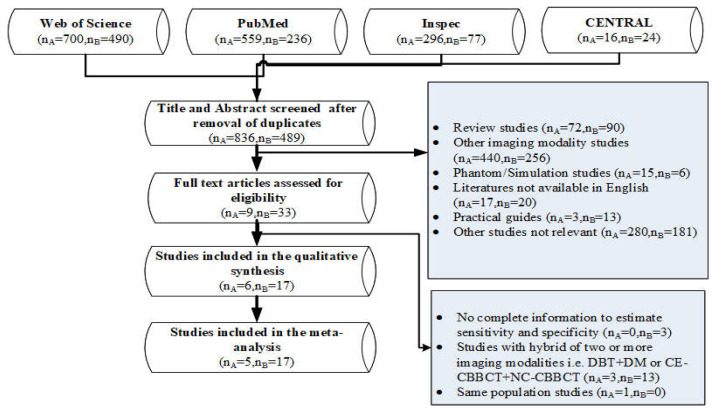
PRISMA flowchart of inclusion and exclusion criteria, $n_{A}$ = number of literature in the CBBCT arm and $n_{B}$ = the number of literature in the DBT arm. PRISMA = Preferred Reporting Items for Systematic Reviews and Meta-analyses. DBT = Digital breast tomosynthesis, DM = Digital mammography, CE-CBBCT = Contrast-Enhanced Cone-beam breast computed tomography, and NC-CBBCT = Non-Contrast Cone-beam breast computed tomography.

PubMed, Inspec, Web of Science and Cochrane Central Register of Controlled Trials (CENTRAL) libraries were searched for relevant literature published from January 2015 up to and including December 2021. We used selected controlled terms extracted from different studies retrieved from each database to build the text words and subject terms as “breast computed tomography”, “Sensitivity”, “Specificity” for the CBBCT arm, and “Digital breast tomosynthesis”, “Sensitivity”, “Specificity” for CBBCT arm and DBT arm, respectively, as shown in the complete PRISMA search path (Figure 1). These selected controlled terms gave a wide representation for the review. In PubMed and CENTRAL databases, selected controlled terms were input as MeSH terms while in the Web of Science and Inspec, we used them as text words for detail see Supplementary File S2.

### 2.2. Eligibility Criteria

Studies were eligible for inclusion in this meta-analysis if they met eligibility criteria adapted from Cochrane diagnostic test accuracy protocol using PRISMA guidelines [24]. Literature was included in the study if it utilized dedicated CBBCT and DBT to detect breast cancer, with at least the sensitivity and specificity reported. The included studies were retrospective, prospective studies, an observer performance study, clinical trials, and comparative studies in different modalities. The exclusion criteria were studies that involved literature reviews, phantom or simulation studies, other radiation studies apart from CBBCT and DBT like radiotherapy and studies with computer-aided detection (CAD), i.e., machine and deep learning application in diagnostic accuracy.

Additionally, a study that reported two or more hybrid modalities like DBT with DM or contrast-enhanced CBBCT (CE-CBBCT) with non-contrast CBBCT (NC-CBBCT) was excluded. However, if it reports both modalities separately, the data for the modality under consideration will be extracted and vice versa. Likewise, for multiple publications that reported the same study or sub-set, the most detailed study in terms of data availability was used.

### 2.3. Study Selection

Articles retrieved for both arms were manually sorted, and duplicates were removed using titles/abstracts, then followed by full text according to the predefined search criteria, and final eligible studies were selected.

### 2.4. Data Collection Process

A standardized extraction sheet was developed, and two independent blinded reviewers (TEK and OAO or CZ and GY) extracted the information needed and resolved the conflict by interrater consensus from eligible studies, which include: study type (prospective or retrospective studies), study clinical settings (diagnostic or screening), number of patients and mean age of the patients, diagnostic equipment model, mean glandular dose, number of radiologists that interpreted the index test and year of experience, sensitivity and specificity. The positive and negative likelihood ratios are computed when they cannot be extracted [25], and other details of formulations of estimated diagnostic test accuracy parameters can be found in [26]. Additionally, the percentage of benign and malignant cases with a brief intervention description is included (Table 1).

### 2.5. Risk of Bias and Quality Appraisal

**Figure 2:**
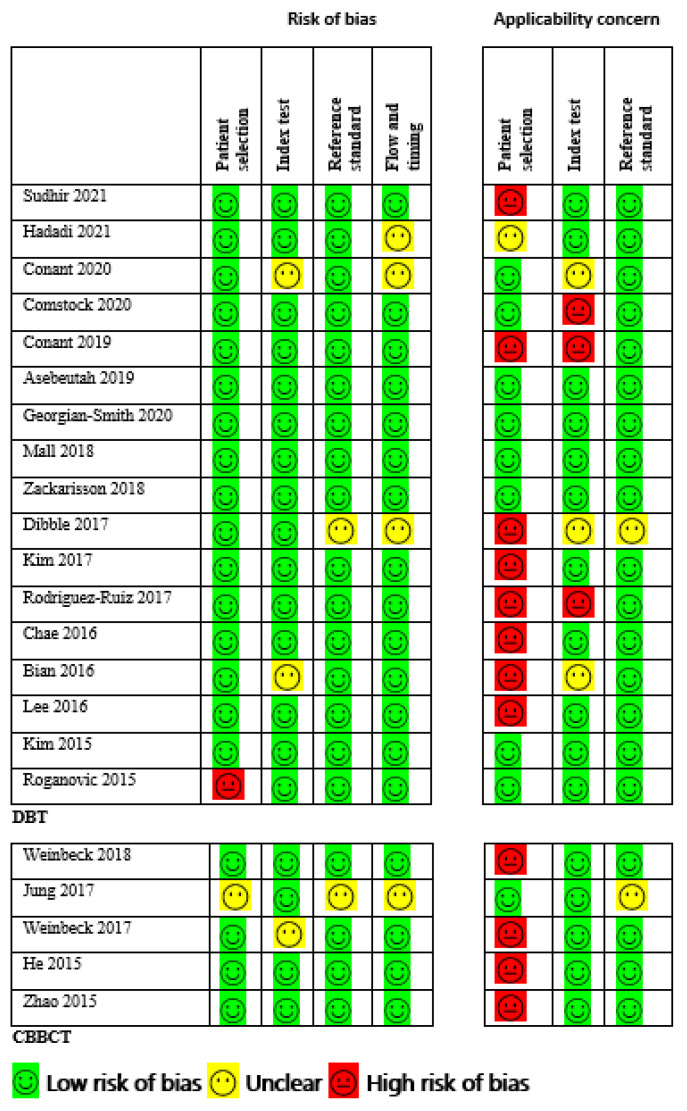
Risk of bias and applicability concerns: reviewers’ judgments about each domain for each included study.

The quality of included studies was assessed using Quality Assessment of Diagnostic Accuracy Studies-Comparative (QUADAS-C), a tool for comparative diagnostic accuracy tests with different cohorts [27], a modified version of QUADAS-2 [28] to ensure appropriateness for comparing the two modalities. The domains assessed were patient selection, index tests, reference standard, flow and timing, and applicability. Two reviewers performed an independent quality assessment, and the final result was based on consensus. The overall study quality is shown in Figure 2.

### 2.6. Data Analysis

A univariate meta-analysis was performed separately for sensitivity and specificity in both CBBCT and DBT to estimate the diagnostic accuracy of each modality using the random-effects model (RE) [29]. The primary outcomes were sensitivity, specificity and summary receiver operating characteristic (SROC) curve. We calculated point estimates and 95% confidence intervals (CI) for each study to ensure consistency in sensitivity and specificity. To plot the SROC curve, we used a bivariate meta-analysis of sensitivity and specificity using R version 4.1.2 with RStudio version 2021.09.1 + 372 implementing “mada” and “meta”, R-packages to estimate the AUC of SROC [30]. Additionally, secondary outcomes like positive likelihood and negative likelihood ratios were estimated using MetaDiSc 1.4 software [31]. Statistical heterogeneity between studies was evaluated with Cochran’s Q test and the *I*^2^ statistic [32]. For the Q statistic, values range 0–40% imply insignificant heterogeneity, 30–60% connote moderate heterogeneity, and 75–100% implies a considerable heterogeneity. Publication bias was evaluated and visualized by constructing a funnel plot [33]. The *p*-values were based on two-sided tests, and the *p*-value < 0.05 was considered statistical significance.

## 3. Results

### 3.1. Study Inclusion

For the DBT arm, a total of 489 different studies were found eligible for abstract screening, 33 studies were checked at full-text (Figure 1). Seventeen studies [10,34,35,36,37,38,39,40,41,42,43,44,45,46,47,48,49] met our inclusion criteria for synthesis and meta-analysis. Additionally, for the CBBCT, 836 different studies were eligible for the title and abstract screening, nine were assessed for full text, and finally, only five studies met our predefined condition [11,12,13,48,49]. The meta-analysis was performed separately using univariate analysis for both CBBCT and DBT. Full details about the inclusion and exclusions criteria are given in the Preferred Items for Systematic Reviews and Meta-Analyses (PRISMA) flowchart (Figure 1).

### 3.2. Overview of Included Studies

For the DBT arm, with 17 studies included, which comprise of retrospective screening studies [34,40,42,44,45,46,48,49,50,51] and prospective studies [35,36,37,38], few prospective clinical trials [10,39], above 95% of all included studies are comparative. All the studies reported sensitivity and specificity, in which the (2 × 2) confusion matrix can be derived, other parameters like positive and negative likelihood ratios and AUC of SROC were estimated using MetaDiSc [31] and “mada” package of R, respectively [30]. Most of the studies specified the total number of benign and malignant lesion cases [10,35,37,38,41,42,43,44,45,46,47]. Approximately 53 % of the studies data were acquired using the Hologic Selenium Dimension model [10,34,36,40,44,45,46,47], 13% goes for Siemens Mammomat Inspiration model [38,39], and 13% also for GE Senographe Essential model [37,42].

The CBBCT arm comprises five studies only, retrospective observers’ studies [12,47], prospective study [48], and retrospective diagnostic study [11]. This majorly consists of comparison studies, i.e., CBBCT vs. DM [12,13], CBBCT vs. DM vs. US, or MRI [11,49]. All the studies reported both the sensitivity and specificity of the diagnostic equipment, while the AUC of SROC was estimated separately like that of the DBT arm. All the studies reported the number of benign and malignant cases, 80% of studies acquired data via the Koning Breast CT (KBCT 1000) model [11,12,13,49].

### 3.3. Quality Assessment and Publication Bias

**Figure 3:**
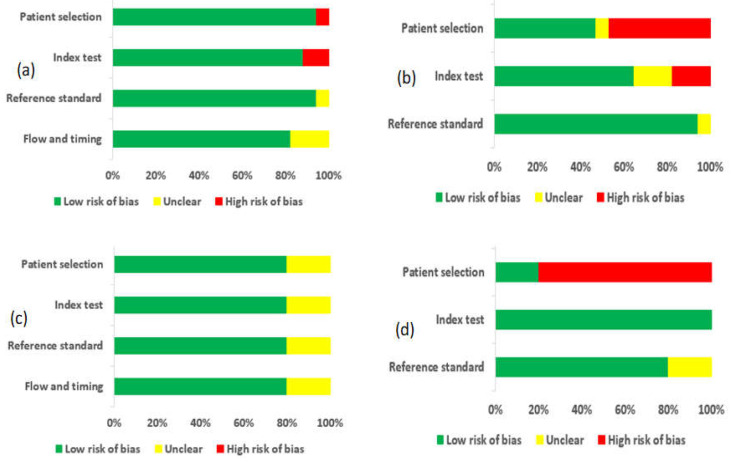
Risk of bias and applicability concerns expressed as percentages across all included studies. (**a**) Risk of bias for DBT; (**b**) Applicability concerns for DBT; (**c**) Risk of bias for CBBCT; (**d**) Applicability concerns for CBBCT.

**Figure 4:**
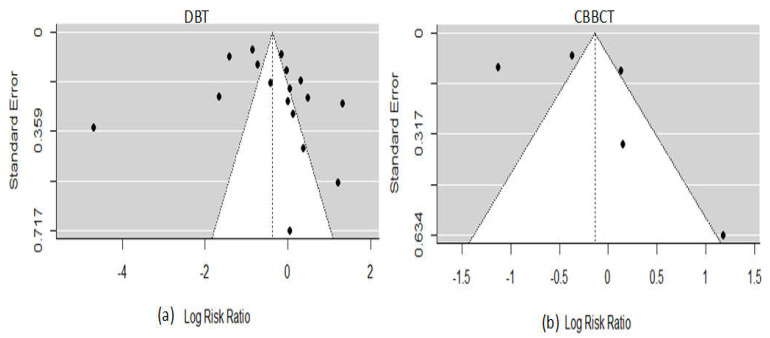
Funnel plots of the likelihood of bias in included studies. (**a**) DBT; (**b**) CBBCT.

In the DBT arm, one study reported a high risk of bias due to inappropriate exclusion and method of patient selection [47]. Two studies (11.8%) reported an unclear risk of bias because the diagnostic threshold was not specified, and no information on whether the readers were blinded to the result of clinical outcomes [34,44]. One study (6.7%) did not give enough information about the pathological findings and, if necessary, follow-up was made, thus providing an unclear risk of bias for a reference standard [40]. Three studies (17.6%) did not give details information if the patients received the reference standard or if the appropriate time interval between the reference standard and index test, thus providing an unclear risk of bias for flow and timing [34,40,51]. Additionally, eight studies (47.1%) had a high risk of bias for applicability concerns regarding patient selection as the criteria for selecting patients did not match exactly our review questions, three studies (17.6%) provided high risk and unclear risk of bias regarding applicability for index test, only one study (5.9%) gave unclear applicability concerns regarding reference standard. The risk of bias and applicability concern and reviewers’ judgment about each domain for all the included study is shown in Figure 2. Likewise, for the CBBCT arm, none of the studies reported a high risk of bias, although the unclear risk of bias exists in patient selection, reference standard, and flow and timing in one study due to scanty information [12,48]. The overview of bias and applicability risk is shown in Figure 3. A visual assessment of funnel plots revealed asymmetrical distribution around inverted funnel for included studies of DBT which signifies publication bias which might be attributed to reporting bias [33], as shown in Figure 4. However, the likelihood of publication bias might also exist in the CBBCT arm due to the small number of studies included in the meta-analysis. More details about the risk of bias and applicability of concerns using QUADASS-2 assessment is shown in Figure 3.

### 3.4. DBT Meta-Analysis

**Figure 5:**
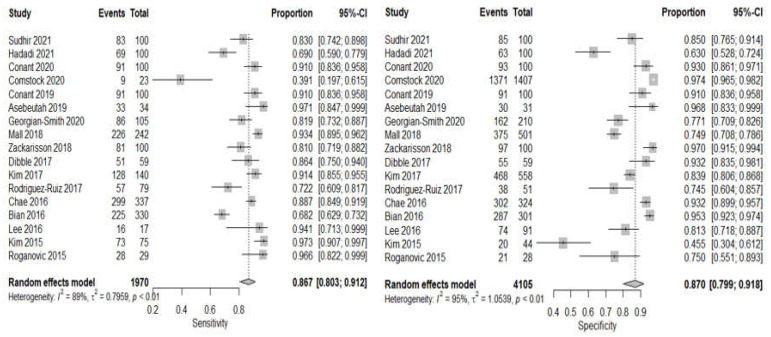
Forest plots using random effect model univariate meta-analysis model for DBT showing pooled sensitivity and pooled specificity.

A total of 17 studies with different observations on sensitivity, specificity, and AUC contributed to the meta-analysis of the DBT arm [10,34,35,36,37,38,39,40,41,42,43,44,45,46,47,48,49]. The forest plot of sensitivity and specificity with point estimates of 95% confidence intervals across different studies are shown in Figure 5. The pooled sensitivity was 86.7% (95% CI: 80.3–91.2, $I^{2}$ = 89) and specificity is 87.0% (95% CI: 79.9–91.8, $I^{2}$ = 95). Since all the within studies had Higgins $I^{2}$ for both sensitivity and specificity above 75%, and the *p*-value of Cochran Q statistic is less than 0.05, which implies there is substantial heterogeneity.

**Figure 6:**
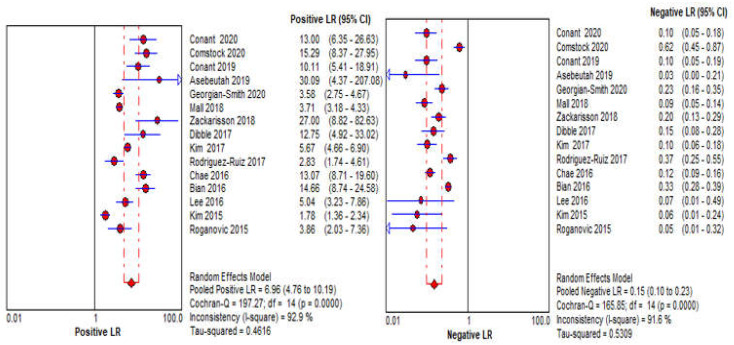
Forest plots of summary of positive (*LR*^+^) and negative (*LR*^−^) likelihood ratios of DBT using random effects bivariate model.

**Figure 7:**
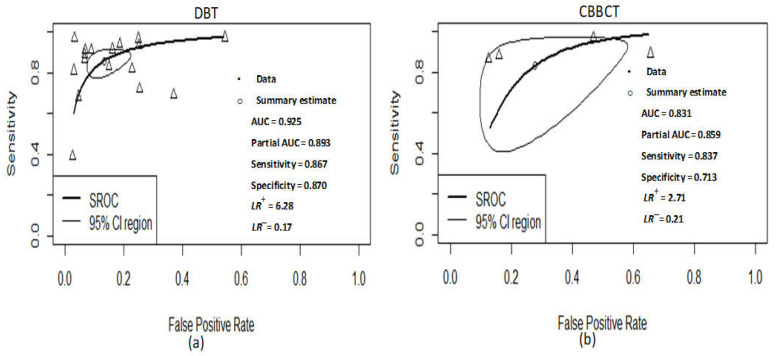
The plot of diagnostic performance using bivariate Summary Receiver Operating Characteristics (SROC) curve. (**a**) SROC of DBT; (**b**) SROC of CBBCT. The prediction region is shown in a dashed dark line, the confidence region shown in a small black ellipse, summary point in black diamond plus ad scaled dataset points for each study in a small triangle. CI: Confidence interval; AUC: area under the curve.

To show both practical and statistical significance between DBT and CBBCT modalities, the difference in sensitivity and specificity of these modalities were estimated, the result of the difference in effect size for sensitivity is 3% (*p*-value = 0.7622) and specificity is 16.4% (*p*-value = 0.0622). The effect size for DBT exceeded CBBCT by 3% and 15.3% for sensitivity and specificity, respectively, which indicate better performance for DBT. Although it is statistically is non-significant since both *p*-values are greater than 0.05. The pooled positive likelihood ratio ($LR^{+}$) is 6.28 (95% CI: 4.40–8.96, $I^{2}$ = 93), while the pooled negative likelihood ratio ($LR^{-}$) is 0.17 (95% CI: 0.12–0.25, $I^{2}$ = 92), as shown in Figure 6. The pooled AUC of SROC is 0.925, as shown in Figure 7a.

### 3.5. CBBCT Meta-Analysis

**Figure 8:**
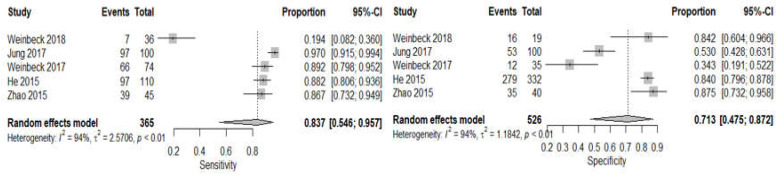
Forest plots using random effects univariate meta-analysis model for CBBCT showing pooled sensitivity and pooled specificity.

**Figure 9:**
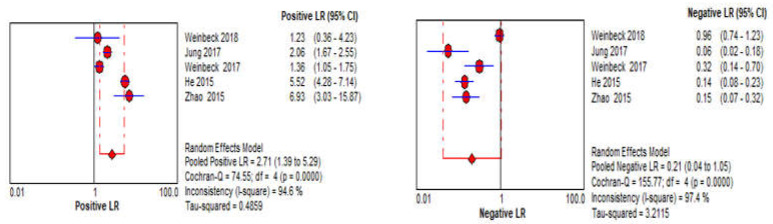
Forest plots of summary of positive and negative likelihood ratios of CBBCT using random effects bivariate model.

A total of five different observation studies were included in the meta-analysis of the CBBCT arm; the summary of all necessary information is tabulated in Table 1. Pooled sensitivity with 95% confidence intervals across the studies is 83.7% (95% CI: 54.6–95.7, $I^{2}$ = 94); while the pooled specificity is 71.3% (95% CI: 47.5–87.2, $I^{2}$ = 94); as shown in Figure 8. There is substantial heterogeneity within studies for both sensitivity and specificity as the value of $I^{2}$ is higher than 75% and a *p*-value less than 0.05. Due to the small number of included studies, further subgroup analyses for evaluating a potential source of heterogeneity were not performed. The pooled positive likelihood ratio ($LR^{+}$) is 2.71 (95% CI: 1.39–5.29, $I^{2}$ = 95), while the pooled negative likelihood ratio ($LR^{-}$) is 0.21 (95% CI: 0.07–0.32, $I^{2}$ = 97), as shown in Figure 9. The pooled AUC of SROC is 0.831, as shown in Figure 7b.

**Table 1:** Characteristics of studies included in digital breast tomosynthesis and cone-beam breast computed tomography.

Study	Country	Equipment	Total No. of Patients	(Mean Age ± SD) Years	No. of Radiol. (Mean Years)	Gland. Dose (mGy)	Sens.	Specf.	Benign Cases (%)	Malig. Cases (%)	Study Intervention
Digital Breast Tomosynthesis											
Sudhir et al. [50]	India	N/A	130	45 ± 12	2 (N/A)	N/A	82.8/100	84.8/100	N/A	N/A	DM vs. DBT vs. US+DBT vs. CEDM ^a^
Hadadi et al. [51]	Australia	N/A	35	N/A	7 (2)	N/A	69/100	63/100	N/A	N/A	DBT vs. DM ^a^
Conant et al. [34]	USA	Hologic Selenia Dimensions	56839	54 ± NA	N/A	N/A	91.2/100	92.6/100	N/A	N/A	DBTvs. DM ^a^
Comstock al. [35]	USA/Germany	N/A	1444	54.9 ± 0.85	2 (N/A)	N/A	9/23	1371/1407	0.6	99.4	One-view DBT vs. DM ^b^
Conant et al. [36]	USA	Hologic Selenia Dimensions	50971	54.6 ± 8.9	13 (N/A)	N/A	90.6/100	91.3/100	N/A	N/A	DBT vs. DM ^b,e^
Asbeutah et al. [37]	Kuwait	GE Senographe Essential	58	54.3 ±12.6	1 (>10)	N/A	33/34	30/31	47.7	52.3	DBT vs. DM ^b,f^
Georgian-Smith et al. [38]	USA	Siemens Mammomat Inspiration system	330	56.3 ± 9.8	31 (4–38)	N/A	86/105	162/210	63.6	31.8	DBT vs. DM ^b,e^
Mall et al. [10]	Australia	Hologic Selenia Dimensions	144	N/A	15 (16)	N/A	226/242	375/501	66.7	33.3	DBT vs. DM ^b,d^
Zackrisson et al. [39]	Sweden	Siemens Mammomat Inspiration system	14848	57.0 ± 10.0	7 (2–14)	2.30	81.1/100	97.2/100	N/A	N/A	DBT vs. DM ^b,d,f^
Dibble et al. [40]	USA	Hologic Selenia Dimensions	59	58.9 ± N/A	3 (6–16)	N/A	51/59	55/59	N/A	N/A	DBT vs. DM ^a^
Kim et al. [41]	Korea	Hologic Selenia Dimensions	698	48.7 ± 11.2	12 (9.3)	1.30	128/140	468/558	79.9	20.1	DBT vs. US ^b,f^
Rodriguez-Ruiz et al. [42]	Netherlands	N/A	181	52 ± N/A	6 (23)	2.41	57/79	38/51	39.2	60.8	DBT vs. DM ^a,f^
Chae et al. [43]	Korea	GE Senographe Essential	319	49.0 ± N/A	3 (8–18)	N/A	299/337	302/324	11.1	88.9	DBT vs. DM ^b,e^
Bian et al. [44]	China	Hologic Selenia Dimensions	631	45.0 ± N/A	3 (3–20)	N/A	225/330	287/301	47.7	52.3	DBT vs. DM ^a^
Lee et al. [45]	Korea	Hologic Selenia Dimensions	108	46.3 ± 7.8	3 (N/A)	1.50	17/17	74/91	84.3	15.7	DBT vs. US ^a,f^
Kim et al. [46]	Korea	Hologic Selenia Dimensions	113	49.6 ± N/A	3 (>13)	N/A	73/75	20/44	37.0	63.0	DBT vs. US ^a,f^
Roganovic et al. [47]	Bosnia and Herzegovina	Hologic Selenia Dimensions	N/A	53.2 ± N/A	1(10)	2.3	29/29	21/28	49.1	50.9	DBT vs. DM vs. MRI ^b,f^
**Cone-Beam Breast Computed Tomography**											
Weinbeck et al. [12]	Germany	Koning (CBCT 1000) Breast CT	41	67.8 ± N/A	2 (>7)	5.85-7.5	7/36	16/19	43.0	51.0	CBBCT vs. MRI vs. DM ^a,e^
Jung et al. [48]	N/A	N/A	30	30 ± N/A	4 (7)	N/A	97/100	53/100	76.5	23.5	CBBCT ^a,c^
Weinbeck et al. [11]	Germany	Koning (CBCT 1000) Breast CT	59	N/A	2 (18.5)	5.8–16.6	66/74	12/35	31.3	66.1	CBBCT vs. DM ^a,c^
He et al. [49]	China	Koning (CBCT 1000) Breast CT	212	48 ± N/A	2 (>10)	8 ± 1.6	97/110	279/332	75.1	24.9	CBBCT vs. DM vs. US ^b^
Zhao et al. [13]	USA	Koning (CBCT 1000) Breast CT	65	55.6 ± 9.8	2 (>7)	5.8–24.84	39/45	35/40	47.1	52.9	CBBCT vs. DM ^b,e^

Note: ^a^ Retrospective study, ^b^ Prospective studies, ^c^ Observer performance studies, ^d^ Clinical trial studies, ^e^ Diagnostic studies, ^f^ Screening studies DBT: Digital Breast Tomosynthesis, DM: Digital Mammography, Sens.—Sensitivity, Specf.—Specificity, Gland. Dose—Mean glandular dose, *LR*^+^: Positive likelihood ratio and *LR*^−^: Negative likelihood ratio, CEDMContrast-enhanced digital mammography.

## 4. Discussion

The systematic review identified 17 studies for the DBT arm and five studies for the CBBCT arm, comparing the diagnostic accuracy using sensitivity, specificity, mean AUC of SROC, positive and negative likelihood ratios as a figure of merits. Our results showed that the pooled sensitivity of DBT was 86.7% (95% CI: 80.3–91.2) and was higher than that of the pooled sensitivity of CBBCT 83.7% (95% CI: 54.6–95.7), with about 3% with a *p*-value of 0.7622. Likewise, the pooled specificity of DBT showed an improvement over CBBCT from 87.7% (95% CI: 79.9–91.8) and 71.3% (95% CI: 47.5–87.2) by 16.4%. The pooled $LR^{+}$ of DBT is 6.28 (95% CI: 4.40–8.96) and was slightly higher than that of CBBCT with pooled $LR^{+}$ of 2.71 (95% CI: 1.39–5.29). The result signifies that DBT is six times more likely to detect patients with breast cancer than patients without breast cancer, as $LR^{+}$ is greater than 10 and $LR^{-}$ is less than 0.1 produces the greatest efficiency [25]. The pooled AUC of SROC of the DBT arm is 0.925 and was significantly higher than that of the CBBCT arm (*p*-value = 0.016), 0.831. The pooled $LR^{+}$ and $LR^{-}$ of the CBBCT are 2.71 and 0.21, respectively, which cause a small change in the pre-test probability [25]. Although the result presented by Uhlig et al. [19] showed a pooled sensitivity of 78.9%, the specificity of 69.7% and AUC of 0.817, the result of our CBBCT arm showed higher improvement in terms of pooled sensitivity and sensitivity and mean AUC value. The summary of pooled results is shown in Table 2.

**Figure A1:**
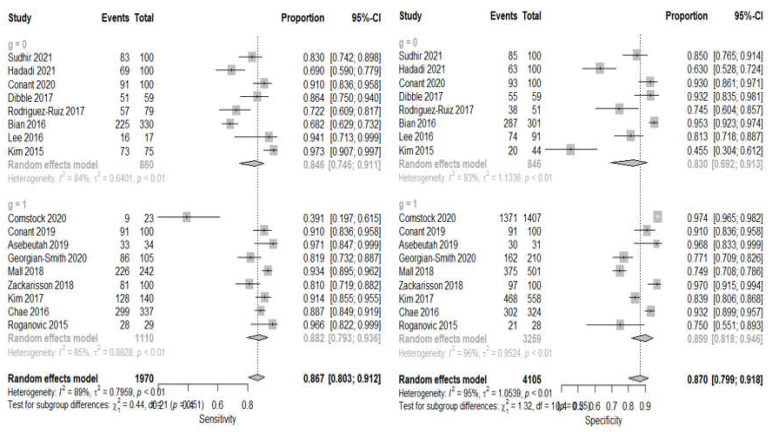
Univariate sub-group analysis of sensitivity and specificity with random model based on the different study protocols. g represents sub-group analysis of data when g = 0 (Retrospective studies) and g = 1 (Prospective studies).

We decided to check the effect of the different study protocols (prospective and retrospective studies) on diagnostic performance by conducting a sub-group analysis. The analysis with retrospective studies has a sensitivity of 84.6% (95% CI: 74.6–91.1, $I^{2}$ = 84% for 8 studies), while that of prospective studies was 86.7% (95% CI: 80.3–91.3, $I^{2}$ = 89% for 9 studies), indicating no significant heterogeneity between the sensitivity as shown in Appendix A (Figure A1). In addition, the specificity is 83.0% (95% CI: 69.2–91.3, $I^{2}$ = 93% for 6 studies) for retrospective studies, while the specificity of prospective studies is 87.0% (95% CI: 79.9–91.8, $I^{2}$ = 96% for 9 studies) in Appendix A (Figure A1). The result indicates that prospective studies of DBT show a slight non-significantly improvement over retrospective studies in terms of sensitivity and specificity with a *p*-value of 0.2509.

This increase in mean AUC of DBT might have resulted from the significantly higher value of sensitivity and specificity recorded by most of the included studies [34,35,36,39,40,42,43,44]. In contrast, similar lower specificity has been recorded in the CBBCT counterparts [12,48,49], contrarily [11,13] reported higher specificity like that of its DBT counterparts as likely supported by Chappell et al. [30], that an effective diagnostic test should have corresponding high sensitivity and specificity, which significantly contribute to the AUC of the SROC curve. The pooled result of our study has demonstrated the diagnostic potency of DBT over the CBBCT for both sensitivity, specificity, positive and negative likelihood ratio, and AUC. When we compared our pooled sensitivity and specificity with that of Belair et al. [20], which had a sensitivity of 87% (95% CI: 80–92) and 70% (95% CI: 60–79) for DBT and CBBCT and specificity of 81% (95% CI: 72–87) and 67% (95% CI: 57–77), we discovered that our pooled sensitivity for the DBT is within the same range, while the pooled specificity has improved by approximately 7.2%. Comparing Belair et al. [20] with our pooled result for CBBCT showed that sensitivity and specificity have improved by 13.7% and 4.3 %, respectively. According to Zuley et al. [21], for lesion visibility and diagnostic accuracy of CBBCT, DBT, and MRI, the AUC of 0.84 and 0.75 was estimated for DBT and CBBCT pooled AUC result improved by 11.3% and 10.8%. The result shows a statistical significance in the pooled AUC for DBT with *p*-value = 0.016, as this will provide better diagnostic power compared to univariate sensitivity and specificity. Although the abbreviated 3D breast MRI has been used to screen patients with a high risk of breast cancer due to its high sensitivity between 80–94% and specificity of 80–100% [52,53], however, some small lesions of less than 5 mm in size and ductal carcinoma in situ (DCIS) are not easily visible due to their diffuse pattern of spread [53,54]. Additionally, the cost of an MRI examination and the time cost for each examination has limited its widespread application [55]. Previous studies on the comparison of CBBCT with DM have shown the higher performance of CBBCT on breast masses characterization [12,13], in cancer detection [48] and improved performance and good interrater agreement among readers [47], therefore making CBBCT a potential modality for improved diagnosis of breast cancer.

The studies have several limitations; firstly, the result of both arms was not extracted from the same studies (comparison with a different cohort) according to Yang et al. [27], as no comparison studies between CBBCT and DBT were available within the study’s scope and range of year covered, which might have introduced a potential bias between the result. Secondly, the sample size of the CBBCT arm is also one-third of that of the DBT arm, the pooled estimate may not fully represent the statistical power we are looking for; thus, the CBBCT result is underrepresented; therefore, the statistical significance of CBBCT might reduce as more sample size tends to increase the statistical significance of a model. Thirdly, due to the recent introduction of CBBCT as a screening or diagnostic imaging modality, no large multicenter prospective or clinical trial studies are available with no standardized acquisition protocol [19], thus making a direct comparison with the DBT modality a daunting task.

## 5. Conclusions

Our study demonstrates that DBT shows improved diagnostic performance over CBBCT with pooled sensitivity, specificity AUC, and positive and negative likelihood ratios. This improvement shows a statistical significance for AUC diagnostic parameter, as this parameter would represent higher diagnostic power compared to its derivative sensitivity and specificity. We believe that the diagnostic performance of CBBCT would continue to improve due to more understanding of the underpinned imaging physics of this modality coupled with computer-aided detection application and better experiences of a radiologist. We recommended more prospective studies on the direct comparison of diagnostic accuracy of CBBCT and DBT for breast cancer characterization and detection.

## Figures and Tables

**Table 2:** Summary of all estimated diagnostic test accuracy.

DOR Parameters	Pooled Value at 95% CI (DBT)	Pooled Value at 95% CI (CBBCT)
Sensitivity	86.7% (80.3–91.2, *I*^2^ = 89%)	83.7% (54.6–95.7 *I*^2^ = 94%)
Specificity	87.0% (79.9–91.8, *I*^2^ = 95%)	71.3% (47.5–87.2, *I*^2^ = 94%)
*LR* ^+^	6.28 (4.40–8.96, *I*^2^ = 93%)	2.71 (1.39–5.29, *I*^2^ = 95%)
*LR* ^−^	0.17 (0.12–0.25, *I*^2^ = 91%)	0.21 (0.04–1.05, *I*^2^ = 97%)
AUC of SROC	0.925	0.831

Note: *LR*^+^ = Positive likelihood ratio, *LR*^−^ = Negative likelihood ratio, DBT = Digital breast tomosynthesis, DM = Digital mammography, CBBCT = Cone-beam breast computed tomography, SROC = Summary Receiver Operating Characteristics, CI = Confidence interval; AUC = area under the curve.

## Data Availability

All the supporting data are included in the study and Appendix A.

## Supplementary Materials

The following are available online at https://www.mdpi.com/article/10.3390/s22093594/s1, File S1: PRISMA checklist table; File S2: Detailed search strategy describing the MeSH and text-word for all the databases.

## Abbreviations

CI: Confidence interval; CBBCT: Cone-beam breast computed tomography; DBT: Digital breast tomosynthesis; DM: Digital mammography; AUC: Area under the curve; $LR^{+}$: Positive likelihood ratio; $LR^{-}$: Negative likelihood ratio; PRISMA: Preferred reporting items for systematic reviews and meta-analyses; QUADAS-2: Quality assessment of diagnostic accuracy studies-2; RE: Random effects.
