# Supplementary material for: Comparison of Diagnostic Test Accuracy of Cone-Beam Breast Computed Tomography and Digital Breast Tomosynthesis for Breast Cancer: A Systematic Review and Meta-Analysis Approach
Source: Sensors (Basel). 2022 May 9;22(9):3594. doi: 10.3390/s22093594 (PMC9101306; doi:10.3390/s22093594)
Supplement: Supplementary file 1 [file sensors-22-03594-s001.zip › sensors-1633086-File S2.pdf]

Table S1. Summary of the description of Medical Subject Headings (MeSH) for the cone-beam breast computed tomography (CBBCT) arm.

| | **Search Terms** |
|---|---|
| #1 | Breast cancer (MeSH);2015-2021 |
| #2 | Sensitivity(MeSH) |
| #3 | Specificity(MeSH) |
| #4 | Cone-beam breast computed tomography(MeSH) |
| #5 | #1**AND**#2**AND**#3**AND**#4 |

Table S2. Summary of the description of Medical Subject Headings (MeSH) for the digital breast tomosynthesis (DBT) arm.

| | **Search Terms** |
|---|---|
| #1 | Breast cancer (MeSH);2015-2021 |
| #2 | Sensitivity(MeSH) |
| #3 | Specificity(MeSH) |
| #4 | Digital breast tomosynthesis (MeSH) **OR** X-ray Breast Tomosynthesis(MeSH) **OR** Breast Tomosynthesis (MeSH) |
| #5 | #1**AND**#2**AND**#3**AND**#4 |

Table S3. Summary of the description of text-word (tw) for the cone-beam breast computed tomography (CBBCT) arm.

| | Search Terms |
|---|---|
| #1 | breast cancer (tw) **OR** breast carcinoma (tw) **OR** breast tumor (tt) **OR** malignant breast tumor (tw) **OR** benign breast tumor 2015-2021 |
| #2 | sensitivity(tw) **OR** [true positive(tw) **AND** false negative(tw)] |
| #3 | specificity(tw) **OR** [true negative(tt) **AND** positive(tt)] |
| #4 | cone-beam breast computed tomography(tw) **OR** dedicated breast computed tomography(tw) **OR** breast CT (tw) |
| #5 | #1**AND**#2**AND**#3**AND**#4 |

Table S4. Summary of the description of text-word (tw) for the digital breast tomosynthesis(DBT) arm.

| | Search Terms |
|---|---|
| #1 | breast cancer (tw) **OR** breast carcinoma (tw) **OR** breast tumor (tw) **OR** malignant breast tumor (tw) **OR** benign breast tumor 2015-2021 |
| #2 | sensitivity(tw) **OR** [true positive(tt) **AND** false negative(tt)] |
| #3 | specificity(tw) **OR** [true negative(tw) **AND** positive(tw)] |
| #4 | digital breast tomosynthesis (tw) **OR** x-ray breast tomosynthesis(tw) **OR** breast tomosynthesis (tw) **OR** 3D mammography |
| #5 | #1**AND**#2**AND**#3**AND**#4 |
